# Defining Core and Penumbra in Ischemic Stroke: A Voxel- and Volume-Based Analysis of Whole Brain CT Perfusion

**DOI:** 10.1038/srep20932

**Published:** 2016-02-10

**Authors:** Yannan Yu, Quan Han, Xinfa Ding, Qingmeng Chen, Keqi Ye, Sheng Zhang, Shenqiang Yan, Bruce C. V. Campbell, Mark W. Parsons, Shaoshi Wang, Min Lou

**Affiliations:** 1 Department of Neurology, Second Affiliated Hospital of Zhejiang University, Hangzhou, Zhejiang, China; 2 Department of Radiology, Second Affiliated Hospital of Zhejiang University, Hangzhou, Zhejiang, China; 3 Department of Medicine and Neurology, Royal Melbourne Hospital, University of Melbourne, Parkville, Australia; 4 Department of Neurology, John Hunter Hospital, and Hunter Medical Research Institute, University of Newcastle, Newcastle, NSW, Australia; 5 Department of Neurology, Shanghai Jiaotong University Affiliated Branch of People’s No. 1 Hospital, Shanghai, China

## Abstract

Whole brain computed tomography perfusion (CTP) has the potential to select eligible patients for reperfusion therapy. We aimed to find the optimal thresholds on baseline CTP for ischemic core and penumbra in acute ischemic stroke. We reviewed patients with acute ischemic stroke in the anterior circulation, who underwent baseline whole brain CTP, followed by intravenous thrombolysis and perfusion imaging at 24 hours. Patients were divided into those with major reperfusion (to define the ischemic core) and minimal reperfusion (to define the extent of penumbra). Receiver operating characteristic (ROC) analysis and volumetric consistency analysis were performed separately to determine the optimal threshold by Youden’s Index and mean magnitude of volume difference, respectively. From a series of 103 patients, 22 patients with minimal-reperfusion and 47 with major reperfusion were included. Analysis revealed delay time ≥ 3 s most accurately defined penumbra (AUC = 0.813; 95% CI, 0.812-0.814, mean magnitude of volume difference = 29.1 ml). The optimal threshold for ischemic core was rCBF ≤ 30% within delay time ≥ 3 s (AUC = 0.758; 95% CI, 0.757-0.760, mean magnitude of volume difference = 10.8 ml). In conclusion, delay time ≥ 3 s and rCBF ≤ 30% within delay time ≥ 3 s are the optimal thresholds for penumbra and core, respectively. These results may allow the application of the mismatch on CTP to reperfusion therapy.

Stroke is the leading cause of death in China. However, only 1.6% of stroke patients receive tissue plasminogen activator (rtPA) therapy in China^1^ despite proven effectiveness in reducing disability. By identifying the tissue at risk (penumbra) and the tissue that is irreversibly injured (ischemic core) for those patients who arrive at hospital outside the time window or with unknown onset time, perfusion imaging may increase the rate of thrombolytic therapy^2^. The presence of a “mismatch” between a small core and large penumbra may indicate the potential for such patients to benefit from off-label reperfusion therapies.

Recent studies focused on computed tomography perfusion (CTP) to select eligible patients for reperfusion therapy^3^,^4^,^5^, with the advantage of fast imaging^6^. However, consensus on the optimal CTP thresholds for penumbra and core has not been reached^7^,^8^,^9^,^10^,^11^. More recently, whole brain CTP has become available, allowing doctors to evaluate the perfusion status throughout the entire brain. Studies have shown that the diagnostic performance of whole brain CTP is considerably different from previous non-whole brain CTP, which does not cover the entire territory of middle cerebral artery (MCA) and could entirely miss lesions in anterior cerebral artery (ACA) territory^7^,^8^,^11^. It is also unclear whether perfusion thresholds are the same when derived from CTP with different coverage of brain voxels. Moreover, most prior studies singly used either receiver operating characteristic (ROC) analysis or volume validation to determine the thresholds^9^,^12^. It may be necessary to perform both methods to derive precise results since they reflect either spatial or volumetric accuracy. In addition, for the Chinese population, only one small study has examined optimal perfusion thresholds^13^. It remains unknown whether the optimal thresholds in the Asian population, with a high incidence of intracranial atherosclerosis, are similar to other populations. We sought to define the specific thresholds of core and penumbra in ischemic stroke based on whole brain CTP, by performing both ROC analysis and volumetric consistency testing. We hypothesized that the infarct area at 24 hours in patients with major reperfusion after thrombolysis could be used to define ischemic core using baseline CTP. The infarct area at 24 hours in patients without reperfusion was used to define the extent of penumbra on baseline CTP. The optimal threshold for core and penumbra was derived from baseline CTP parameters by dual-verification of voxel- and volume-base analysis in our consecutively treated thrombolysis patients.

## Materials and Methods

### Ethics statement

Written informed consent was obtained from each patient or an appropriate family member. The human ethics committee of The Second Affiliated Hospital of Zhejiang University approved the protocol of this study. All clinical investigations were conducted according to the principles expressed in the Declaration of Helsinki.

### Patients

We reviewed 457 consecutive patients with acute ischemic stroke within 6 hours of onset between July 2011 and March 2015. All received baseline imaging evaluation and then were treated with intravenous rtPA. The inclusion criteria: 1) stroke onset ≤6 hours; 2) patients with National Institute of Health Stroke Scale (NIHSS) scores ≥ 4; 3) underwent CTP before intravenous rtPA, and CTP or MRP at 24 hours after thrombolysis; 4) confirmed as anterior-circulation ischemic stroke. Exclusion criteria: 1) baseline hypoperfusion (Tmax  ≥  6 s) volume ≤ 10 ml^14^; 2) patients with unknown symptom onset time; 3) hemorrhagic transformation that hindered the evaluation of imaging; 4) image quality that was insufficient for analysis.

### Imaging protocol

CTP was performed on a dual-source 64-slice CT scanner (SOMATOM Definition Flash; Siemens, Forchheim, Germany), including non-enhanced CT head scan (120 kV, 320 mA, contiguous 5 mm axial slices), and volume perfusion CT (VPCT) (100 mm in the z-axis, 4 seconds delay after start of contrast medium injection, 74.5 seconds total imaging duration, 80 kV, 120 mA, effective dose = 3.68 mSv, slice thickness 10 mm, collimation 32 × 1.2 mm). VPCT consisted of 26 consecutive spiral acquisitions of brain. All 26 scans were divided into 4 parts: 1) 2 scans with 3 s cycle time; 2) 15 scans with 1.5 s cycle time; 3) 4 scans with 3 s cycle time; 4) 5 scans with 6 s cycle time. A 60-mL bolus of contrast medium (Iopamidol; Braccosine, Shanghai, China) was used at a flow rate of 6 ml/s, followed by a 20 mL saline chaser at 6 ml/s. 4D CTA images were reconstructed from VPCT in axial, coronal and sagittal planes with 20-mm-thick MIPS.

MRI on a 3.0T system (Sigma Excite HD, General Electric, Milwaukee, USA) equipped with an 8-channel phased array head coil. Foam pads were inserted into the space between the subject’s head and the MRI head coil to minimize head motion. The MRI protocol included diffusion weighted imaging (DWI), enhanced 3D multi-echo GRE T2*-weighted angiography (ESWAN), and PWI. DWI was performed with a spin echo-planar sequence (field of view = 240 mm, slice thickness = 1 mm, number of slices = 18, slice gap = 1 mm, acquisition matrix = 160 × 160). PWI was performed with gradient echo-planar imaging (field of view = 240 mm, repetitive time = 1500 ms, echo time = 30 ms, acquisition matrix = 128 × 128. Repetitive scanning times = 50. gadolinium dose = 15 ml, contrast speed = 4–5 ml/s, duration = l min 15 s).

### Image analysis

CTP and MRI were analyzed by commercial software MIStar (Apollo Medical Imaging Technology, Melbourne, Australia). The software automatically generated delay time, absolute cerebral blood flow (CBF), cerebral blood volume (CBV), and mean transit time (MTT) maps using delay- and dispersion-corrected singular value deconvolution (dd-SVD)^15^. The relative CBF (rCBF), relative CBV (rCBV), relative MTT (rMTT) were calculated based on the automatically derived normal values.

**Figure 1:**
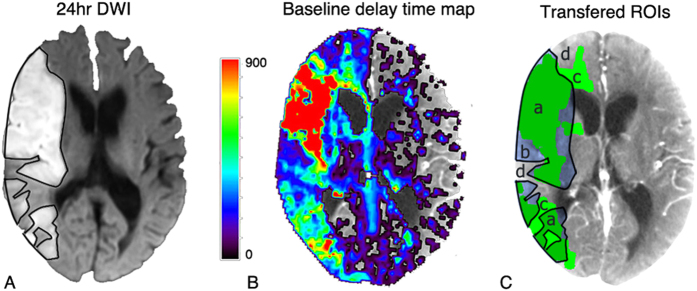
An example of image processing for voxel-based analysis. Images have been co-registered and re-sliced. (**A**) Black lines on 24 hr DWI represent the ROIs of the 24 hr infarct lesion. (**B**) baseline delay time map. MIStar automatically marked set threshold as green region (delay time ≥ 3 s was shown as green regions in **C**). (**C**) 24 hr infarction ROIs were saved and co-registered to baseline CT. Then, every threshold was marked and analyzed. a, areas thought to be “true positive”; b, areas of “false negative”; c, areas of “false positive”; d, areas of “true negative”.

Non-contrast CT or DWI at 24 hours was co-registered and re-sliced to reach the maximum correspondence with baseline CTP (using MIStar). Infarct lesions on co-registered and re-sliced image were manually delineated by an experienced neurologist (5 years experience) using the MIStar ROI tool. These ROIs were drawn without reference to other images except for baseline non-contrast CT to exclude old infarct, and then transferred to baseline CT perfusion maps for voxel-based analysis (Fig. 1). A range of thresholds were investigated (Table 1). (Supplemental material)

### Patient groups

Since Tmax ≥ 4~6 s has been demonstrated to be comparable between CTP and MRP^16^,^17^,^18^,^19^,^20^, in defining hypoperfused regions, we used it to calculate the reperfusion rate: Reperfusion rate = 1 – [24 h Tmax ≥ 6 s volume / baseline Tmax ≥ 6 s volume]. We defined 3 patient groups: minimal-reperfusion ( ≤ 20% reperfusion), partial reperfusion (20~80% reperfusion), and major reperfusion ( ≥ 80% reperfusion)^8^,^21^. The minimal-reperfusion group were used to define the extent of penumbra, as the corresponding infarct area at 24 h represented the sum of baseline penumbra and core. The major reperfusion group were used to define the extent of core, as the infarct area at 24 h should correspond to baseline ischemic core.

### Statistical analysis

Statistical analysis was performed using SPSS 17.0 (SPSS Inc, Chicago, USA), R software (R Development Core Team, R: A Language Environment for Statistical Computing, Vienna, Austria; ISBN 3-900051-07-0; http://www.R-project.org, 2011).

### Voxel-based analysis

In voxel-based analysis, voxels that fell within both infarct area at 24 hours and baseline hypoperfusion lesion (delineated by the thresholds in Table 1) were considered as “true positive”; voxels not within infarct area but in baseline hypoperfusion lesion as “false positive”; voxels within infarct area but not in baseline hypoperfusion lesion as “false negative”; and voxels not within both lesions were “true negative”. (Figure 1) ROC curve analysis was then performed and the optimal threshold was determined by Youden’s index. To reduce the false-positive regions from leukoaraiosis in analysis of ischemic core^20^,^22^, only voxels within penumbra was analyzed, once the penumbral threshold was identified.

### Volume-based analysis

Volume-based analysis was conducted by volumetric agreement between baseline threshold-based hypoperfusion lesion and infarct area at 24 hours with Bland-Altman and correlation analysis. The optimal threshold should have the best volumetric agreement, determined by the smallest mean magnitude (absolute) difference in lesion volume, see supplemental material).

## Results

### Baseline clinical data

In total, 154 patients treated with intravenous rtPA had both baseline CTP and perfusion imaging at 24 hours, of whom 129 had anterior-circulation ischemic stroke. Twenty-six patients were excluded due to three reasons: baseline hypoperfusion region ≤10 ml (n = 16), hemorrhagic transformation that hindered the evaluation (n = 6), and poor image quality (n = 4).

Ultimately 103 patients were included in the analysis, of whom 27 (26.2%) patients had minimal-reperfusion and 55 (53.4%) had major reperfusion. Twenty-one (20.4%) patients with partial reperfusion were not analyzed further. At 24 hours, CTP was performed in 44 patients and MRP in 59 patients. Clinical data is listed in Table 2.

### Defining ischemic penumbra

**Figure 2:**
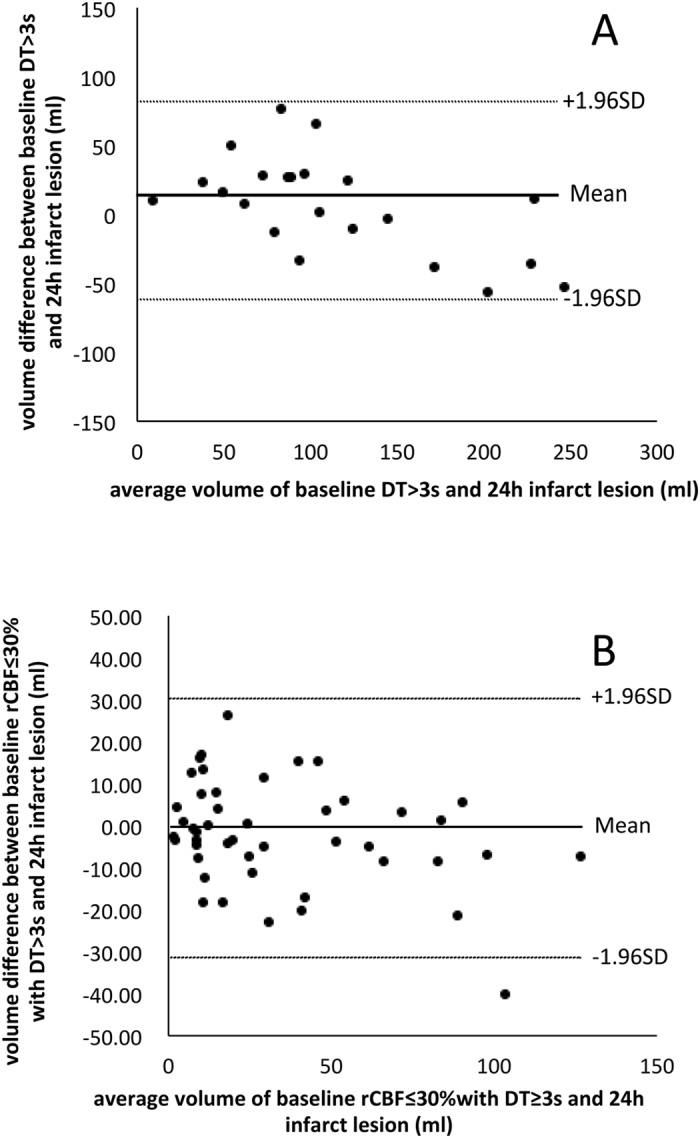
Volumetric agreement of baseline delay time ≥ 3 s and 24 h infarct in non-reperfusers (penumbral threshold) and the core threshold using rCBF ≤ 30% within delay time ≥ 3 s versus 24 h infarct lesion in those with major reperfusion. Solid lines represent mean difference (bias) and dashed lines for 95% limits of agreement. (**A**) Bland-Altman plot between baseline delay time ≥ 3 s and 24 h infarct lesion. (**B**) Bland-Altman plot between baseline rCBF ≤ 30% within delay time ≥ 3 s and 24 h infarct lesion.

Of 27 patients with minimal-reperfusion, 22 were ultimately included after 5 patients were excluded due to brain herniation (n = 3) and new infarct (n = 2) at 24 h that hampered the calculation of final infarct volume. The optimal thresholds for each parameter and the Bland-Altman plot are listed in Table 3 and Figure 2. ROC analysis demonstrated that the optimal parameter was delay time (AUC = 0.813; 95% CI, 0.812-0.814) with the threshold of delay time ≥ 3 s (Youden’s Index = 0.49, sensitivity = 0.75, specificity = 0.74). Delay time ≥ 3 s also had the best agreement and correlation in volume-based analysis (mean magnitude of volume difference = 29.1 ml).

### Defining ischemic core

Among 55 patients with major reperfusion, 47 were included after exclusion of 8 patients where the infarct lesion on non-contrast CT at 24 hours was too indistinct and small to define. The performance of other parameters and Bland-Altman plot of rCBF are detailed in Table 4 and Figure 2. ROC analysis indicated that the optimal parameter was rCBF (AUC = 0.758; 95% CI, 0.757-0.760) with an optimal threshold of rCBF ≤ 30% within area of delay time ≥ 3 s (Youden’s Index = 0.40, sensitivity = 0.64, specificity = 0.76). The volumetric analysis also revealed that rCBF ≤ 30% was the optimal threshold for ischemic core (mean magnitude of volume difference = 10.8 ml).

## Discussion

In this study, by performing both ROC analysis and volumetric consistency testing, we confirmed that delay time ≥ 3 s was the optimal threshold for ischemic penumbra and rCBF ≤ 30% within the area of delay time ≥ 3 s was the optimal threshold for ischemic core. To the best of our knowledge, this is the first calculation of specific thresholds for ischemic core and penumbra using whole brain CTP in the Chinese population.

Based on our results, delay time ≥ 3 s was found to be the optimal threshold for penumbra, with a high Youden’s Index and strong volumetric agreement. This result is slightly different from a previous study in Caucasian patients, which revealed that the optimal thresholds for penumbra were delay time ≥ 2 s based on non-whole brain CTP analysis^8^. In our study, delay time ≥ 2 s had a high sensitivity, but tended to overestimate penumbra volume. Delay time is a specific parameter generated by MIStar using ddSVD method. It is similar to Tmax but has correction of both delay and dispersion which may better reflect the pathophysiological process of the time for contrast to travel from selected AIF ROI to the tissue voxel^8^,^23^,^24^.

Delay time was also superior to other parameters for defining penumbra, in contrast to some previous studies which used rMTT^9^. Very limited studies of Chinese stroke patients suggested that rMTT ≥ 150% was the optimal threshold for penumbra^13^,^25^. Our data also indicated that rMTT ≥ 150% reasonably predicted penumbra, but we found that the recommended threshold varied with different post-processing algorithms^26^. Moreover, unlike delay time, MTT is not delay- and dispersion- corrected. This could explain the large mean magnitude of volumetric difference using MTT/rMTT, indicating overestimation and instability in predicting penumbra. CBF was also suggested as the optimal threshold for penumbra previously^27^. However, in our study CBF was less reliable than delay time with low Youden’s Index and large mean magnitude of volume difference, mainly because the measurement of CBF was easily influenced by the presence of leukoaraiosis, and the CBF in grey and white matter was different. CBV/rCBV was not a good predictor of penumbra, as the auto-regulation in penumbra tissue recruits collaterals and dilates vessels, generally leading to normal or increased CBV value.

Our optimal threshold for ischemic core was rCBF ≤ 30% (constrained within the region of delay time ≥ 3 s). An unconstrained rCBF threshold tends to include “false-positive” regions of leukoaraiosis, reducing its accuracy in delineating ischemic core^20^,^22^. Some prior studies indicated that CBV or CBF plus CBV can define ischemic core^9^,^10^,^27^,^28^,^29^. However, rCBV and CBV had poorer performance in our study than rCBF, which was consistent with recent studies^7^,^15^, possibly because we used a wider range of rCBF thresholds (20~80%) than prior studies (i.e. 40–90%^9^) and different post-processing methods. A previous Chinese study suggested delay time ≥ 2 s was the optimal threshold for ischemic core^13^. However, we found that they used CT perfusion maps at 24 hours to determine the threshold of ischemic core, which could be inaccurate. We observed that in some cases, regions of hypoperfused tissue at baseline were irreversibly injured despite reperfusion at 24 hours. Therefore defining core using CTP at 24 hours may underestimate the extent of infarction.

Our study had three strengths. First, we used whole brain CTP. Previous non-whole brain CTP mainly covered voxels in basal ganglia and cortex (MCA territory), so its thresholds could only be validated under the same imaging protocol. With a wider scan range, the whole brain CTP covered more white matter and ACA territory and we then included patients with occlusion of ACA or M2 segment or M2 beyond. Therefore, our results could be more widely applied. Second, we used dd-SVD algorithm, which has theoretical advantages in adjusting for the effects of delay and dispersion seen with collateral flow^15^. It is widely accepted that different processing algorithms have a major impact on the results of optimal thresholds. Third, we used two independent and complementary statistical approaches, with both voxel- and volume-based analysis. ROC analysis can evaluate the spatial accuracy, but is strongly influenced by the chosen of reference region, while the Bland-Altman plot and correlation study assess the volumetric accuracy, but ignore the spatial correspondence. We believe the combination of these two methods is useful to cross-validate the optimal perfusion thresholds.

The limitations of this study include the small number of enrolled cases and retrospective analysis from a single center. Second, either CTP or MRP were used to define reperfusion. However, we chose Tmax, which has been demonstrated to have comparable values on both CTP and MRP^19^,^20^, to define the perfusion lesion, which may minimize the difference in hypoperfusion volume. Third, we used non-contrast CT or DWI at 24 hours, which may potentially introduce variability in infarct volume delineation. Therefore, we performed ROC analysis separately and found similar results in patients with non-contrast CT and MRP at 24 hours. (See supplemental material) Fourth, the use of reperfusion at 24 hours to divide groups may include patients who had late reperfusion within 24 hours. The exact time of reperfusion after intravenous thrombolysis is unknown. Therefore, any infarct growth between imaging and reperfusion would lead to an increase in the optimal rCBF threshold for core. Further studies in patients receiving thrombectomy may be more suitable to validate the optimal thresholds. Fifth, compared with MRI, CT scan involves ionising radiation. However, the rapid acquisition and availability of CT have cemented its role in acute ischemic stroke imaging protocols. Moreover, we did not investigate clinical outcome variation between imaging strategies, which would warrant further prospective study.

### Summary

Based on whole brain CTP and dual-verification of voxel- and volume-based analysis, we firstly confirmed that delay time ≥ 3 s and rCBF ≤ 30% constrained within the region of delay time ≥ 3 s were the optimal thresholds for ischemic penumbra and core. Since reperfusion or non-reperfusion was used to verify these thresholds, we believe these results may allow the application of the “mismatch” to reperfusion therapy in clinical trials and practice.

## Additional Information

**How to cite this article**: Yu, Y. *et al*. Defining Core and Penumbra in Ischemic Stroke: A Voxel- and Volume-Based Analysis of Whole Brain CT Perfusion. *Sci. Rep.* **6**, 20932; doi: 10.1038/srep20932 (2016).

## Supplementary Material

Supplementary Information

## Figures and Tables

**Table 1:** Range and increments for parameters to investigate the threshold.

Parameters	Range	Increment
Delay time (s)	1~10	1
rCBF (%)*	20~55(60~80)	5(10)
CBF (ml/100 g/min)	2~20	2
rCBV (%)	20~80	10
CBV (ml/100 g)	0.5~5.0	0.5
rMTT (%)	125~250	25
MTT (s)	5~15	2

*Increment of 5% was applied in the range of 20~55% where the threshold is likely to be based on previous studies.

CBF, cerebral blood flow; CBV, cerebral blood volume; MTT, mean transient time.

**Table 2:** Baseline clinical data.

	Minimal-reperfusion group (n = 27)	Major reperfusion group (n = 55)	*P* value
Female, %	8 (29.6)	30 (54.5)	0.033
Median age, y (range)	74 (46, 91)	70 (21, 88)	0.877
Median baseline NIHSS (range)	13 (5, 27)	11 (0, 32)	0.272
24 hr Infarct volume, ml (*x* ± *s*)	109.1 ± 76.6	35.5 ± 34.4	< 0.001
DNT, min (*x* ± *s*)	72 ± 29	74 ± 39	0.92
ONT, min (*x* ± *s*)	253 ± 118	208 ± 76	0.047

NIHSS, National Institutes of Health Stroke Scale; DNT, door to needle time; ONT, onset to needle time.

**Table 3:** Accuracy of optimal thresholds for penumbra (minimal-reperfusion group).

Threshold	Youden’s Index	Sensitivity	Specificity	Mean magnitude (ml)	r	P value
Delay time ≥ 2 s	0.45	0.83	0.61	44.9	0.849	< 0.001
Delay time ≥ 3 s	0.49	0.75	0.74	29.1	0.900	< 0.001
Delay time ≥ 4 s	0.49	0.66	0.83	30.5	0.879	< 0.001
rMTT ≥ 150%	0.45	0.73	0.72	49.4	0.631	0.002
MTT ≥ 9 s	0.43	0.74	0.68	69.0	0.584	0.004
rCBF ≤ 50%	0.38	0.64	0.74	41.8	0.732	0.001
CBF ≤ 10 ml/100 g/min	0.36	0.57	0.79	50.1	0.691	< 0.001
rCBV ≤ 60%	0.18	0.44	0.74	61.8	0.400	0.101
CBV ≤ 1.5 ml/100 g	0.17	0.44	0.73	62.0	0.300	0.121

More data in supplemental material.

CBF, cerebral blood flow; CBV, cerebral blood volume; MTT, mean transient time.

**Table 4:** Accuracy of optimal thresholds for ischemic core (major reperfusion group).

Threshold (within delay time ≥ 3 s)	Youden’s Index	Sensitivity	Specificity	Mean magnitude (ml)	r	P value
rCBF ≤ 30%	0.40	0.64	0.76	10.8	0.881	< 0.001
CBF ≤ 6 ml/100 g/min	0.36	0.55	0.81	19.4	0.716	< 0.001
rCBV ≤ 50%	0.27	0.49	0.78	24.7	0.530	< 0.001
CBV ≤ 1 ml/100 g	0.26	0.40	0.86	22.5	0.566	< 0.001
Delay time ≥ 7 s	0.28	0.57	0.71	17.6	0.752	< 0.001
rMTT ≥ 200%	0.20	0.53	0.66	18.4	0.743	< 0.001
MTT ≥ 13 s	0.20	0.41	0.79	27.0	0.348	0.017

More data in supplemental material.

CBF, cerebral blood flow; CBV, cerebral blood volume; MTT, mean transient time.
