# Supplementary material for: Defining Core and Penumbra in Ischemic Stroke: A Voxel- and Volume-Based Analysis of Whole Brain CT Perfusion
Source: Sci Rep. 2016 Feb 10;6:20932. doi: 10.1038/srep20932 (PMC4748242; doi:10.1038/srep20932)
Supplement: Supplementary Information [file srep20932-s1.pdf]

Online Supplement

Defining Core and Penumbra in Ischemic Stroke: A Voxel- and Volume-Based Analysis of Whole Brain CT Perfusion

Yannan Yu, MD; Quan Han, MD; Xinfa Ding, MD; Qingmeng Chen, MD; Keqi Ye, MD; Sheng Zhang, MD, PhD; Shenqiang Yan, MD; Bruce C.V. Campbell, MBBS, BMedSc, PhD, FRACP; Mark W. Parsons, MBBS, PhD, FRACP; Shaoshi Wang, MD; *Min Lou, MD, PhD

Supplemental methods

MIStar (Apollo Medical Imaging Technology, Melbourne, Australia) automatically selected a normal major artery (usually the ACA or MCA) for arterial input function (AIF), and a large draining vein (such as the sagittal sinus) for venous outflow function (VOF). Deconvolution of the tissue enhancement curve and AIF was performed using delay- and dispersion-corrected singular value deconvolution (dd-SVD). Non-brain voxels, such as ventricle and skull, were removed automatically according to Hounsfield unit threshold (usually set as 25 HU for ventricle and 150 HU for skull). Vessel segmentation threshold was set as Voxels with CBF value≥2×(normal CBF value).

Twenty-four hour image was co-registered to the baseline CTP manually, using rigid body transformation with or without scaling and affine, and were re-sliced to reach the maximum correspondence of the baseline CTP and 24h image (using MIStar). ROIs of Infarct lesions on co-registered and re-sliced 24h image were delineated for each slice, and then the total infarct lesion volumes for one case were then calculated by MIStar.

In voxel-based analysis, to prevent a very large number of true negative voxels from overwhelming the ratio to false positive voxels when calculating specificity, only the hemispheric brain voxels were analyzed for penumbra threshold.

In volume-based analysis, the corresponding regions of investigated thresholds were automatically delineated and marked on baseline CTP, and then each baseline perfusion lesion volume was calculated by MIStar. Mean magnitude was used as a determinant index for threshold evaluation. Mean magnitude refers to the average of absolute mean difference values. It can be a more reasonable index to define the best agreement than mean difference because it reflects both bias and standard deviation.

Supplementary Tables

Supplemental table I. The volumetric analysis of minimal-reperfusion group (penumbra)

Thresholds	Mean magnitude (ml)	Mean difference (ml)	95%LoA		r	P value
			Lower limit (ml)	Upper limit (ml)		
DT>1s	76.2	75.7	-21.4	172.7	0.761	<0.001
DT>2s	44.9	36.1	-42.4	114.7	0.849	<0.001
DT>3s	29.1	7.1	-62.6	76.8	0.900	<0.001
DT>4s	30.5	-15.6	-94.4	63.3	0.879	<0.001
DT>5s	42.4	-35.6	-117.0	45.7	0.875	<0.001
DT>6s	54.2	-49.7	-138.1	38.6	0.861	<0.001
DT>7s	64.1	-60.8	-157.4	35.8	0.849	<0.001
DT>8s	72.6	-70.0	-172.9	32.9	0.837	<0.001
DT>9s	79.8	-77.8	-188.1	32.6	0.817	<0.001
DT>10s	85.9	-84.5	-202.0	33.0	0.790	<0.001
rCBF<20%	87.9	-87.9	-204.8	29.0	0.744	<0.001
rCBF<25%	77.9	-77.9	-185.0	29.2	0.784	<0.001
rCBF<30%	69.1	-67.1	-175.1	40.9	0.755	<0.001
rCBF<35%	59.5	-52.9	-158.5	52.7	0.755	<0.001
rCBF<40%	52.9	-36.1	-151.0	78.8	0.671	0.001
rCBF<45%	43.8	-18.9	-126.5	88.8	0.715	<0.001
rCBF<50%	44.9	-1.3	-116.8	114.3	0.647	0.001
rCBF<55%	44.8	22.5	-83.7	128.7	0.708	<0.001
rCBF<60%	56.9	35.9	-82.8	154.6	0.614	0.002
rCBF<70%	77.4	72.1	-45.3	189.5	0.625	0.002
rCBF<80%	105.8	105.1	-17.2	227.5	0.589	0.004
CBF<2ml/100g/min	104.0	-104.0	-242.3	34.4	0.755	<0.001
CBF<4ml/100g/min	93.5	-93.5	-216.6	29.7	0.715	<0.001
CBF<6ml/100g/min	80.1	-78.4	-196.6	39.7	0.681	<0.001
CBF<8ml/100g/min	63.9	-56.5	-169.3	56.2	0.691	<0.001
CBF<10ml/100g/min	50.1	-22.5	-135.7	90.7	0.662	0.001

CBF<12ml/100g/min	59.7	19.0	-163.4	201.4	0.400	0.107
CBF<14ml/100g/min	65.1	47.1	-123.7	217.8	0.425	0.049
CBF<16ml/100g/min	91.4	83.1	-89.5	255.6	0.480	0.024
CBF<18ml/100g/min	119.8	114.3	-66.4	295.0	0.445	0.038
CBF<20ml/100g/min	136.2	133.8	-38.5	306.1	0.487	0.025
rCBV<20%	90.2	-88.2	-210.9	34.6	0.746	<0.001
rCBV<30%	93.4	-93.4	-212.3	25.5	0.778	<0.001
rCBV<40%	82.2	-81.1	-198.5	36.2	0.720	<0.001
rCBV<50%	71.6	-59.8	-191.6	72.0	0.484	0.023
rCBV<60%	61.8	-32.6	-177.6	112.4	0.400	0.101
rCBV<70%	63.6	-1.3	-161.5	158.8	0.300	0.237
rCBV<80%	71.1	27.4	-139.2	194.1	0.200	0.264
CBV<0.5ml/100g	100.0	-100.0	-229.3	29.2	0.714	<.001
CBV<1ml/100g	86.2	-84.6	-201.9	32.7	0.726	<0.001
CBV<1.5ml/100g	62.0	-36.1	-174.7	102.4	0.400	0.054
CBV<2ml/100g	67.9	30.8	-126.8	188.4	0.300	0.121
CBV<2.5ml/100g	101.6	91.8	-68.4	252.1	0.400	0.082
CBV<3ml/100g	147.4	146.8	-25.8	319.4	0.400	0.109
CBV<3.5ml/100g	172.2	172.2	15.5	328.9	0.465	0.029
CBV<4ml/100g	196.9	196.9	39.5	354.3	0.445	0.038
CBV<4.5ml/100g	205.9	205.9	51.2	360.6	0.491	0.020
CBV<5ml/100g	224.3	224.3	68.6	380.0	0.455	0.033
rMTT>125%	110.6	110.6	-28.3	249.6	0.585	0.004
rMTT>150%	49.4	29.7	-105.6	164.9	0.631	0.002
rMTT>175%	45.2	-20.6	-131.7	90.6	0.685	<0.001
rMTT>200%	62.1	-56.3	-172.0	59.4	0.674	0.001
rMTT>225%	84.1	-84.1	-216.4	48.3	0.608	0.003
rMTT>250%	97.9	-97.9	-242.1	46.3	0.400	0.053
MTT>5s	241.6	241.6	94.5	388.7	0.523	0.012
MTT>7s	154.9	154.9	-3.2	312.9	0.608	0.003
MTT>9s	69.0	48.2	-122.3	218.8	0.584	0.004
MTT>11s	49.8	-22.3	-156.2	111.6	0.583	0.004
MTT>13s	69.8	-66.6	-191.5	58.4	0.569	0.006

MTT>15s	86.8	-86.8	-215.7	42.1	0.566	0.006

Supplemental table II. The volumetric analysis of major reperfusion group (ischemic core)

Thresholds	Mean magnitude (ml)	Mean difference (ml)	95% LoA		r	P value
			Lower limit (ml)	Upper limit (ml)		
DT>1s	96.3	96.3	-1.0	193.7	0.573	<0.001
DT>2s	66.7	66.5	-17.2	150.2	0.663	<0.001
DT>3s	47.6	46.4	-27.0	119.7	0.721	<0.001
DT>4s	32.2	28.9	-32.9	90.7	0.761	<0.001
DT>5s	23.2	15.2	-38.3	68.7	0.776	<0.001
DT>6s	19.2	4.4	-43.1	51.9	0.775	<0.001
DT>7s	17.6	-4.6	-50.6	41.3	0.752	<0.001
DT>8s	17.3	-11.3	-52.8	30.2	0.788	<0.001
DT>9s	19.5	-16.3	-59.5	26.9	0.769	<0.001
DT>10s	22.3	-20.6	-66.8	25.6	0.737	<0.001
rCBF<20%	20.3	-19.1	-57.6	19.5	0.861	<0.001
rCBF<25%	16.4	-12.8	-47.2	21.6	0.865	<0.001
rCBF<30%	10.8	-0.4	-32.5	31.6	0.881	<0.001
rCBF<35%	18.4	13.7	-30.3	57.7	0.784	<0.001
rCBF<40%	34.5	34.0	-14.7	82.7	0.777	<0.001
rCBF<45%	50.9	50.9	-4.7	106.5	0.723	<0.001
rCBF<50%	69.9	69.9	2.8	137.0	0.682	<0.001
rCBF<55%	85.5	85.5	12.3	158.7	0.621	<0.001
rCBF<60%	104.3	104.3	25.8	182.8	0.606	<0.001
rCBF<70%	139.2	139.2	51.7	226.7	0.536	<0.001
rCBF<80%	172.4	172.4	76.2	268.6	0.463	0.001
CBF<2ml/100g/min	31.6	-31.6	-90.3	27.0	0.602	<0.001
CBF<4ml/100g/min	24.4	-24.1	-72.5	24.3	0.728	<0.001
CBF<6ml/100g/min	19.4	-9.5	-58.0	39.0	0.716	<0.001
CBF<8ml/100g/min	28.4	13.5	-60.4	87.4	0.555	<0.001
CBF<10ml/100g/min	45.5	40.8	-49.6	131.2	0.466	0.001
CBF<12ml/100g/min	80.0	78.7	-71.3	228.8	0.249	0.092

CBF<14ml/100g/min	106.7	106.6	-46.3	259.5	0.252	0.088
CBF<16ml/100g/min	134.7	134.7	-28.8	298.2	0.217	0.143
CBF<18ml/100g/min	160.3	160.3	-10.6	331.3	0.198	0.182
CBF<20ml/100g/min	186.5	186.5	2.8	370.2	0.133	0.372
rCBV<20%	30.8	-30.7	-85.9	24.6	0.686	<0.001
rCBV<30%	28.1	-26.7	-77.5	24.0	0.747	<0.001
rCBV<40%	21.2	-13.3	-63.6	37.0	0.671	<0.001
rCBV<50%	24.7	10.9	-49.1	70.8	0.530	<0.001
rCBV<60%	45.0	41.4	-30.7	113.6	0.432	0.002
rCBV<70%	78.2	76.5	-14.5	167.5	0.347	0.017
rCBV<80%	110.8	109.9	9.0	210.8	0.314	0.032
CBV<0.5ml/100g	30.4	-30.2	-84.6	24.1	0.682	<0.001
CBV<1ml/100g	22.5	-12.9	-68.7	42.8	0.566	<0.001
CBV<1.5ml/100g	52.3	44.5	-55.6	144.5	0.258	0.080
CBV<2ml/100g	112.7	112.1	-24.4	248.7	0.179	0.228
CBV<2.5ml/100g	167.2	167.2	17.9	316.5	0.113	0.451
CBV<3ml/100g	206.2	206.2	56.5	356.0	0.118	0.429
CBV<3.5ml/100g	232.6	232.6	86.7	378.6	0.070	0.641
CBV<4ml/100g	253.2	253.2	113.3	393.0	0.083	0.580
CBV<4.5ml/100g	264.6	264.6	126.1	403.1	0.052	0.729
CBV<5ml/100g	275.1	275.1	139.3	410.9	0.071	0.636
rMTT>125%	144.3	144.3	21.9	266.8	0.615	<0.001
rMTT>150%	67.4	67.1	-33.2	167.3	0.722	<0.001
rMTT>175%	31.9	27.4	-51.3	106.2	0.720	<0.001
rMTT>200%	18.4	5.5	-51.3	62.3	0.743	<0.001
rMTT>225%	19.5	-13.9	-67.4	39.6	0.624	<0.001
rMTT>250%	26.1	-24.1	-81.5	33.3	0.522	<0.001
MTT>5s	296.6	296.6	174.9	418.3	0.140	0.348
MTT>7s	180.4	180.4	10.5	350.2	0.297	0.042
MTT>9s	85.3	83.4	-84.8	251.6	0.364	0.012
MTT>11s	42.0	30.5	-107.3	168.3	0.324	0.026
MTT>13s	27.0	-1.2	-94.1	91.8	0.348	0.017

MTT>15s	26.8	-20.0	-89.2	49.1	0.310	0.034

Supplemental table III. ROC analysis results of minimal-reperfusion group

Thresholds	Sensitivity	Specificity	Youden's index
DT>1s	0.91	0.39	0.30
DT>2s	0.83	0.61	0.45
DT>3s	0.75	0.74	0.49
DT>4s	0.66	0.83	0.49
DT>5s	0.58	0.88	0.46
DT>6s	0.50	0.92	0.42
DT>7s	0.42	0.94	0.36
DT>8s	0.35	0.96	0.31
DT>9s	0.29	0.97	0.26
DT>10s	0.23	0.98	0.21
rCBF<20%	0.25	0.98	0.23
rCBF<25%	0.34	0.96	0.30
rCBF<30%	0.41	0.93	0.34
rCBF<35%	0.47	0.89	0.36
rCBF<40%	0.53	0.85	0.38
rCBF<45%	0.59	0.79	0.38
rCBF<50%	0.64	0.74	0.38
rCBF<55%	0.69	0.69	0.38
rCBF<60%	0.73	0.63	0.36
rCBF<70%	0.80	0.53	0.33
rCBF<80%	0.85	0.43	0.28
CBF<2ml/100g/min	0.08	1.00	0.08
CBF<4ml/100g/min	0.21	0.98	0.20
CBF<6ml/100g/min	0.35	0.94	0.29
CBF<8ml/100g/min	0.47	0.88	0.34
CBF<10ml/100g/min	0.57	0.79	0.36
CBF<12ml/100g/min	0.65	0.70	0.36
CBF<14ml/100g/min	0.73	0.62	0.34
CBF<16ml/100g/min	0.78	0.53	0.31
CBF<18ml/100g/min	0.83	0.45	0.28

CBF<20ml/100g/min	0.86	0.38	0.24
rCBV<20%	0.10	0.98	0.08
rCBV<30%	0.16	0.96	0.12
rCBV<40%	0.25	0.9	0.15
rCBV<50%	0.34	0.83	0.17
rCBV<60%	0.44	0.74	0.18
rCBV<70%	0.52	0.65	0.17
rCBV<80%	0.61	0.57	0.18
CBV<0.5ml/100g	0.11	0.98	0.09
CBV<1ml/100g	0.26	0.89	0.15
CBV<1.5ml/100g	0.44	0.73	0.17
CBV<2ml/100g	0.61	0.55	0.16
CBV<2.5ml/100g	0.75	0.38	0.12
CBV<3ml/100g	0.84	0.24	0.09
CBV<3.5ml/100g	0.91	0.14	0.06
CBV<4ml/100g	0.96	0.07	0.03
CBV<4.5ml/100g	0.99	0.02	0.01
CBV<5ml/100g	1.00	0.01	0.00
rMTT>125%	0.87	0.47	0.34
rMTT>150%	0.73	0.72	0.45
rMTT>175%	0.55	0.86	0.41
rMTT>200%	0.39	0.93	0.32
rMTT>225%	0.26	0.97	0.23
rMTT>250%	0.16	0.98	0.14
MTT>5s	0.98	0.07	0.04
MTT>7s	0.90	0.36	0.26
MTT>9s	0.74	0.68	0.43
MTT>11s	0.53	0.87	0.40
MTT>13s	0.34	0.95	0.28
MTT>15s	0.19	0.98	0.17

Supplemental table IV. ROC analysis results of major-reperfusion group

Thresholds	Sensitivity	Specificity	Youden's index
DT>1s	0.99	0.02	0.01
DT>2s	0.96	0.07	0.03
DT>3s	0.92	0.15	0.07
DT>4s	0.85	0.31	0.16
DT>5s	0.75	0.48	0.23
DT>6s	0.66	0.61	0.27
DT>7s	0.57	0.71	0.28
DT>8s	0.48	0.79	0.27
DT>9s	0.40	0.85	0.25
DT>10s	0.33	0.89	0.22
rCBF<20%	0.46	0.90	0.36
rCBF<25%	0.56	0.84	0.39
rCBF<30%	0.64	0.76	0.40
rCBF<35%	0.71	0.68	0.39
rCBF<40%	0.76	0.60	0.36
rCBF<45%	0.80	0.53	0.33
rCBF<50%	0.83	0.47	0.30
rCBF<55%	0.86	0.41	0.27
rCBF<60%	0.88	0.36	0.24
rCBF<70%	0.92	0.27	0.19
rCBF<80%	0.94	0.20	0.14
CBF<2ml/100g/min	0.17	0.98	0.15
CBF<4ml/100g/min	0.38	0.91	0.29
CBF<6ml/100g/min	0.55	0.81	0.36
CBF<8ml/100g/min	0.66	0.69	0.36
CBF<10ml/100g/min	0.74	0.59	0.33
CBF<12ml/100g/min	0.80	0.49	0.29
CBF<14ml/100g/min	0.84	0.41	0.25
CBF<16ml/100g/min	0.88	0.34	0.21
CBF<18ml/100g/min	0.90	0.28	0.18

CBF<20ml/100g/min	0.92	0.23	0.15
rCBV<20%	0.15	0.97	0.12
rCBV<30%	0.25	0.94	0.19
rCBV<40%	0.38	0.87	0.25
rCBV<50%	0.49	0.78	0.27
rCBV<60%	0.59	0.68	0.27
rCBV<70%	0.69	0.56	0.25
rCBV<80%	0.76	0.47	0.22
CBV<0.5ml/100g	0.17	0.96	0.14
CBV<1ml/100g	0.40	0.86	0.26
CBV<1.5ml/100g	0.61	0.66	0.27
CBV<2ml/100g	0.76	0.47	0.23
CBV<2.5ml/100g	0.85	0.31	0.16
CBV<3ml/100g	0.91	0.19	0.10
CBV<3.5ml/100g	0.95	0.11	0.06
CBV<4ml/100g	0.97	0.06	0.03
CBV<4.5ml/100g	0.99	0.02	0.01
CBV<5ml/100g	1.00	0.01	0.00
rMTT>125%	0.94	0.17	0.10
rMTT>150%	0.85	0.33	0.18
rMTT>175%	0.65	0.56	0.21
rMTT>200%	0.53	0.66	0.20
rMTT>225%	0.37	0.79	0.16
rMTT>250%	0.27	0.85	0.12
MTT>5s	0.98	0.05	0.03
MTT>7s	0.92	0.20	0.12
MTT>9s	0.77	0.43	0.20
MTT>11s	0.58	0.65	0.23
MTT>13s	0.41	0.79	0.20
MTT>15s	0.25	0.88	0.13

Supplemental table V. separate ROC analysis results of minimal-reperfusion group (24hr MRP)

Thresholds	Sensitivity	Specificity	Youden's index
DT>1s	0.91	0.41	0.31
DT>2s	0.81	0.62	0.44
DT>3s	0.72	0.75	0.47
DT>4s	0.63	0.84	0.47
DT>5s	0.55	0.89	0.44
DT>6s	0.47	0.93	0.40
DT>7s	0.39	0.95	0.34
DT>8s	0.32	0.97	0.29
DT>9s	0.27	0.98	0.25
DT>10s	0.22	0.99	0.20
rCBF<20%	0.22	0.99	0.21
rCBF<25%	0.31	0.97	0.28
rCBF<30%	0.39	0.93	0.32
rCBF<35%	0.45	0.90	0.34
rCBF<40%	0.50	0.85	0.35
rCBF<45%	0.56	0.80	0.37
rCBF<50%	0.62	0.75	0.37
rCBF<55%	0.67	0.70	0.37
rCBF<60%	0.70	0.64	0.34
rCBF<70%	0.78	0.54	0.32
rCBF<80%	0.84	0.44	0.28
CBF<2ml/100g/min	0.09	1.00	0.09
CBF<4ml/100g/min	0.23	0.98	0.21
CBF<6ml/100g/min	0.37	0.94	0.31
CBF<8ml/100g/min	0.49	0.87	0.35
CBF<10ml/100g/min	0.58	0.78	0.37
CBF<12ml/100g/min	0.67	0.69	0.36
CBF<14ml/100g/min	0.73	0.60	0.34
CBF<16ml/100g/min	0.79	0.52	0.31

CBF<18ml/100g/min	0.83	0.44	0.27
CBF<20ml/100g/min	0.87	0.36	0.23
rCBV<20%	0.12	0.98	0.10
rCBV<30%	0.18	0.96	0.14
rCBV<40%	0.27	0.90	0.17
rCBV<50%	0.36	0.83	0.19
rCBV<60%	0.45	0.74	0.19
rCBV<70%	0.54	0.65	0.19
rCBV<80%	0.61	0.56	0.18
CBV<0.5ml/100g	0.12	0.98	0.10
CBV<1ml/100g	0.28	0.88	0.16
CBV<1.5ml/100g	0.46	0.72	0.18
CBV<2ml/100g	0.63	0.53	0.16
CBV<2.5ml/100g	0.75	0.37	0.12
CBV<3ml/100g	0.84	0.24	0.08
CBV<3.5ml/100g	0.91	0.14	0.05
CBV<4ml/100g	0.95	0.08	0.03
CBV<4.5ml/100g	0.98	0.03	0.02
CBV<5ml/100g	1.00	0.01	0.00
rMTT>125%	0.87	0.46	0.33
rMTT>150%	0.75	0.68	0.42
rMTT>175%	0.53	0.87	0.40
rMTT>200%	0.40	0.93	0.33
rMTT>225%	0.24	0.97	0.21
rMTT>250%	0.16	0.99	0.15
MTT>5s	0.98	0.07	0.04
MTT>7s	0.90	0.36	0.26
MTT>9s	0.73	0.67	0.41
MTT>11s	0.51	0.87	0.38
MTT>13s	0.33	0.95	0.27
MTT>15s	0.18	0.98	0.17

Supplemental table VI. Separate ROC analysis of minimal-reperfusion group (24hr CTP, penumbra)

Thresholds	Sensitivity	Specificity	Youden's index
DT>1s	0.92	0.37	0.29
DT>2s	0.86	0.60	0.46
DT>3s	0.80	0.72	0.52
DT>4s	0.72	0.80	0.52
DT>5s	0.64	0.87	0.51
DT>6s	0.55	0.90	0.45
DT>7s	0.47	0.93	0.40
DT>8s	0.39	0.95	0.34
DT>9s	0.32	0.96	0.28
DT>10s	0.26	0.97	0.23
rCBF<20%	0.23	0.98	0.21
rCBF<25%	0.32	0.96	0.28
rCBF<30%	0.40	0.92	0.32
rCBF<35%	0.47	0.88	0.35
rCBF<40%	0.54	0.83	0.37
rCBF<45%	0.59	0.78	0.37
rCBF<50%	0.65	0.72	0.37
rCBF<55%	0.70	0.67	0.37
rCBF<60%	0.74	0.61	0.35
rCBF<70%	0.81	0.51	0.32
rCBF<80%	0.87	0.40	0.27
CBF<2ml/100g/min	0.04	1.00	0.04
CBF<4ml/100g/min	0.16	0.99	0.16
CBF<6ml/100g/min	0.30	0.97	0.27
CBF<8ml/100g/min	0.43	0.91	0.34
CBF<10ml/100g/min	0.53	0.83	0.37
CBF<12ml/100g/min	0.62	0.75	0.36
CBF<14ml/100g/min	0.69	0.66	0.34

CBF<16ml/100g/min	0.74	0.57	0.31
CBF<18ml/100g/min	0.80	0.48	0.28
CBF<20ml/100g/min	0.84	0.40	0.24
rCBV<20%	0.08	0.98	0.06
rCBV<30%	0.14	0.96	0.10
rCBV<40%	0.23	0.90	0.13
rCBV<50%	0.32	0.84	0.16
rCBV<60%	0.43	0.75	0.18
rCBV<70%	0.52	0.67	0.19
rCBV<80%	0.61	0.57	0.18
CBV<0.5ml/100g	0.06	0.99	0.05
CBV<1ml/100g	0.19	0.94	0.13
CBV<1.5ml/100g	0.36	0.81	0.17
CBV<2ml/100g	0.53	0.64	0.17
CBV<2.5ml/100g	0.69	0.46	0.15
CBV<3ml/100g	0.81	0.31	0.12
CBV<3.5ml/100g	0.89	0.20	0.08
CBV<4ml/100g	0.94	0.11	0.05
CBV<4.5ml/100g	0.97	0.05	0.02
CBV<5ml/100g	1.00	0.01	0.00
rMTT>125%	0.87	0.49	0.36
rMTT>150%	0.75	0.72	0.47
rMTT>175%	0.59	0.85	0.44
rMTT>200%	0.43	0.91	0.34
rMTT>225%	0.29	0.95	0.24
rMTT>250%	0.18	0.97	0.15
MTT>5s	0.98	0.06	0.04
MTT>7s	0.90	0.37	0.27
MTT>9s	0.76	0.71	0.47
MTT>11s	0.56	0.87	0.43
MTT>13s	0.36	0.95	0.31

MTT>15s	0.20	0.97	0.17

Supplemental table VII. Separate ROC analysis results of major reperfusion group (24hr MRI, ischemic core)

Thresholds	Sensitivity	Specificity	Youden's index
DT>1s	0.98	0.03	0.01
DT>2s	0.95	0.07	0.02
DT>3s	0.91	0.15	0.05
DT>4s	0.83	0.31	0.14
DT>5s	0.73	0.48	0.22
DT>6s	0.63	0.61	0.24
DT>7s	0.53	0.71	0.25
DT>8s	0.44	0.80	0.23
DT>9s	0.36	0.86	0.22
DT>10s	0.29	0.91	0.20
rCBF<20%	0.42	0.92	0.33
rCBF<25%	0.51	0.86	0.37
rCBF<30%	0.61	0.78	0.39
rCBF<35%	0.68	0.70	0.38
rCBF<40%	0.74	0.60	0.34
rCBF<45%	0.77	0.54	0.31
rCBF<50%	0.81	0.47	0.28
rCBF<55%	0.84	0.42	0.26
rCBF<60%	0.87	0.37	0.24
rCBF<70%	0.90	0.28	0.19
rCBF<80%	0.93	0.21	0.14
CBF<2ml/100g/min	0.17	0.98	0.14
CBF<4ml/100g/min	0.38	0.92	0.30
CBF<6ml/100g/min	0.54	0.82	0.36
CBF<8ml/100g/min	0.65	0.71	0.36
CBF<10ml/100g/min	0.72	0.60	0.33
CBF<12ml/100g/min	0.78	0.50	0.28
CBF<14ml/100g/min	0.82	0.42	0.25
CBF<16ml/100g/min	0.86	0.35	0.21

CBF<18ml/100g/min	0.88	0.30	0.18
CBF<20ml/100g/min	0.91	0.25	0.16
rCBV<20%	0.16	0.97	0.13
rCBV<30%	0.26	0.94	0.20
rCBV<40%	0.39	0.87	0.26
rCBV<50%	0.51	0.77	0.28
rCBV<60%	0.61	0.65	0.26
rCBV<70%	0.69	0.54	0.23
rCBV<80%	0.76	0.44	0.20
CBV<0.5ml/100g	0.18	0.97	0.15
CBV<1ml/100g	0.42	0.85	0.27
CBV<1.5ml/100g	0.63	0.64	0.27
CBV<2ml/100g	0.77	0.44	0.21
CBV<2.5ml/100g	0.85	0.30	0.15
CBV<3ml/100g	0.91	0.19	0.10
CBV<3.5ml/100g	0.94	0.12	0.06
CBV<4ml/100g	0.97	0.06	0.03
CBV<4.5ml/100g	0.99	0.03	0.01
CBV<5ml/100g	1.00	0.00	0.00
rMTT>125%	0.91	0.22	0.14
rMTT>150%	0.82	0.40	0.21
rMTT>175%	0.64	0.62	0.26
rMTT>200%	0.52	0.73	0.25
rMTT>225%	0.35	0.84	0.19
rMTT>250%	0.24	0.89	0.13
MTT>5s	0.98	0.04	0.02
MTT>7s	0.89	0.24	0.13
MTT>9s	0.70	0.48	0.18
MTT>11s	0.52	0.69	0.20
MTT>13s	0.36	0.82	0.18
MTT>15s	0.21	0.91	0.12

Supplemental table VIII. Separate ROC analysis results of major reperfusion group (24hr CTP, ischemic core)

Thresholds	Sensitivity	Specificity	Youden's index
DT>1s	0.99	0.02	0.01
DT>2s	0.97	0.07	0.04
DT>3s	0.93	0.16	0.09
DT>4s	0.86	0.31	0.17
DT>5s	0.78	0.47	0.24
DT>6s	0.69	0.60	0.29
DT>7s	0.60	0.70	0.31
DT>8s	0.52	0.78	0.30
DT>9s	0.45	0.83	0.28
DT>10s	0.38	0.87	0.25
rCBF<20%	0.41	0.89	0.30
rCBF<25%	0.53	0.82	0.35
rCBF<30%	0.61	0.75	0.36
rCBF<35%	0.69	0.66	0.35
rCBF<40%	0.75	0.60	0.34
rCBF<45%	0.79	0.53	0.32
rCBF<50%	0.83	0.46	0.29
rCBF<55%	0.86	0.40	0.26
rCBF<60%	0.88	0.35	0.23
rCBF<70%	0.92	0.25	0.17
rCBF<80%	0.94	0.18	0.12
CBF<2ml/100g/min	0.17	0.97	0.14
CBF<4ml/100g/min	0.38	0.90	0.29
CBF<6ml/100g/min	0.56	0.79	0.35
CBF<8ml/100g/min	0.67	0.68	0.35
CBF<10ml/100g/min	0.76	0.57	0.33
CBF<12ml/100g/min	0.82	0.48	0.29
CBF<14ml/100g/min	0.86	0.39	0.25
CBF<16ml/100g/min	0.89	0.32	0.21

CBF<18ml/100g/min	0.92	0.26	0.18
CBF<20ml/100g/min	0.94	0.21	0.14
rCBV<20%	0.14	0.96	0.10
rCBV<30%	0.23	0.93	0.16
rCBV<40%	0.36	0.88	0.23
rCBV<50%	0.48	0.80	0.28
rCBV<60%	0.57	0.71	0.28
rCBV<70%	0.68	0.60	0.28
rCBV<80%	0.75	0.50	0.25
CBV<0.5ml/100g	0.16	0.96	0.12
CBV<1ml/100g	0.37	0.87	0.24
CBV<1.5ml/100g	0.59	0.70	0.29
CBV<2ml/100g	0.74	0.51	0.25
CBV<2.5ml/100g	0.84	0.34	0.18
CBV<3ml/100g	0.91	0.21	0.12
CBV<3.5ml/100g	0.95	0.12	0.07
CBV<4ml/100g	0.97	0.07	0.04
CBV<4.5ml/100g	0.99	0.03	0.02
CBV<5ml/100g	1.00	0.00	0.00
rMTT>125%	0.97	0.26	0.22
rMTT>150%	0.92	0.41	0.33
rMTT>175%	0.76	0.59	0.35
rMTT>200%	0.65	0.67	0.33
rMTT>225%	0.48	0.79	0.27
rMTT>250%	0.37	0.84	0.22
MTT>5s	0.99	0.05	0.04
MTT>7s	0.95	0.15	0.10
MTT>9s	0.84	0.37	0.22
MTT>11s	0.65	0.59	0.24
MTT>13s	0.45	0.75	0.21
MTT>15s	0.28	0.85	0.13

Supplemental table IX. ROC analysis of each parameter (minimal-reperfusion group)

Parameter	AUC	95%CI	
		Lower bound	Upper bound
DT	0.813	0.812	0.814
rCBF	0.762	0.761	0.763
CBF	0.752	0.751	0.753
rCBV	0.625	0.623	0.626
CBV	0.626	0.625	0.627
rMTT	0.782	0.781	0.783
MTT	0.760	0.759	0.761

Supplemental table X. ROC analysis of each parameter (major reperfusion group)

Parameter	AUC	95%CI	
		Lower bound	Upper bound
DT	0.680	0.678	0.681
rCBF	0.758	0.757	0.76
CBF	0.735	0.733	0.736
rCBV	0.682	0.681	0.684
CBV	0.683	0.681	0.685
rMTT	0.639	0.636	0.643
MTT	0.653	0.652	0.655

Supplementary Figures

Figures I

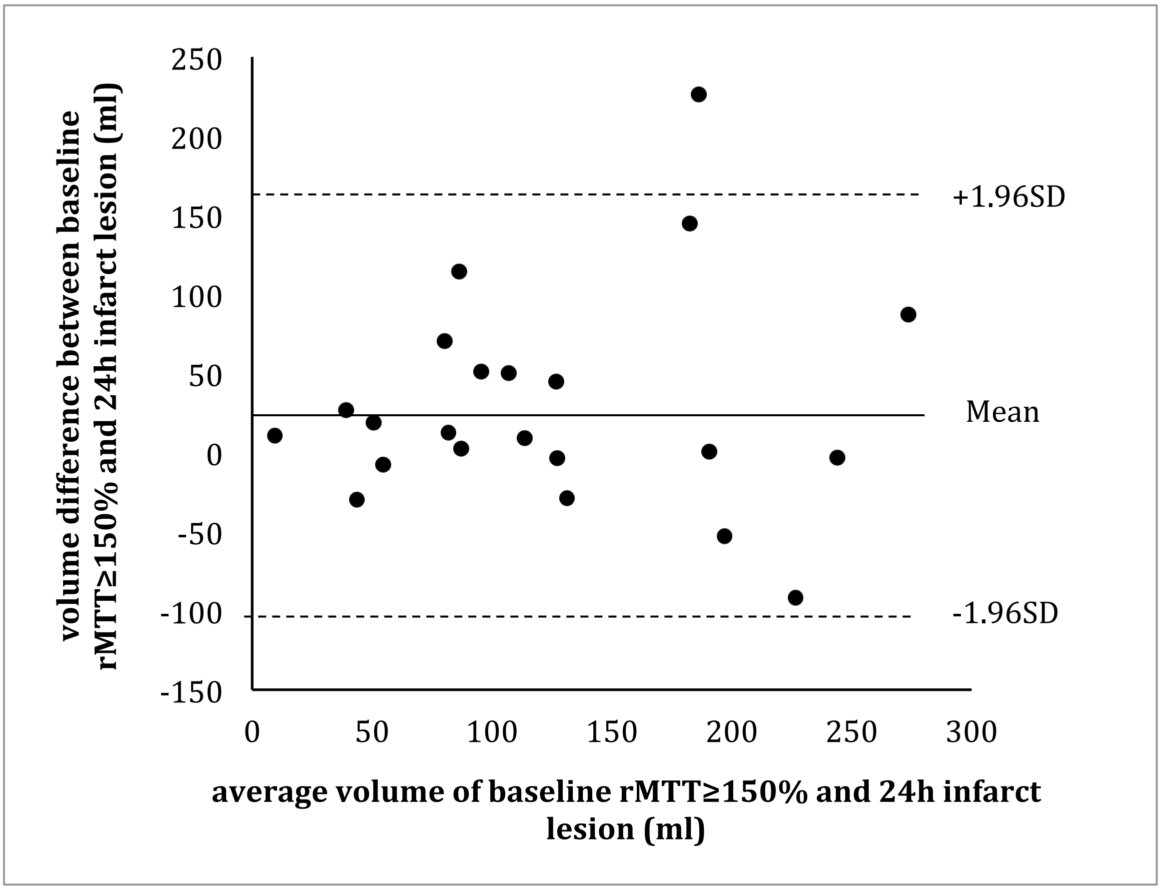

Figure I. Volumetric agreement of baseline rMTT≥150% (minimal-reperfusion group). the

Figure II

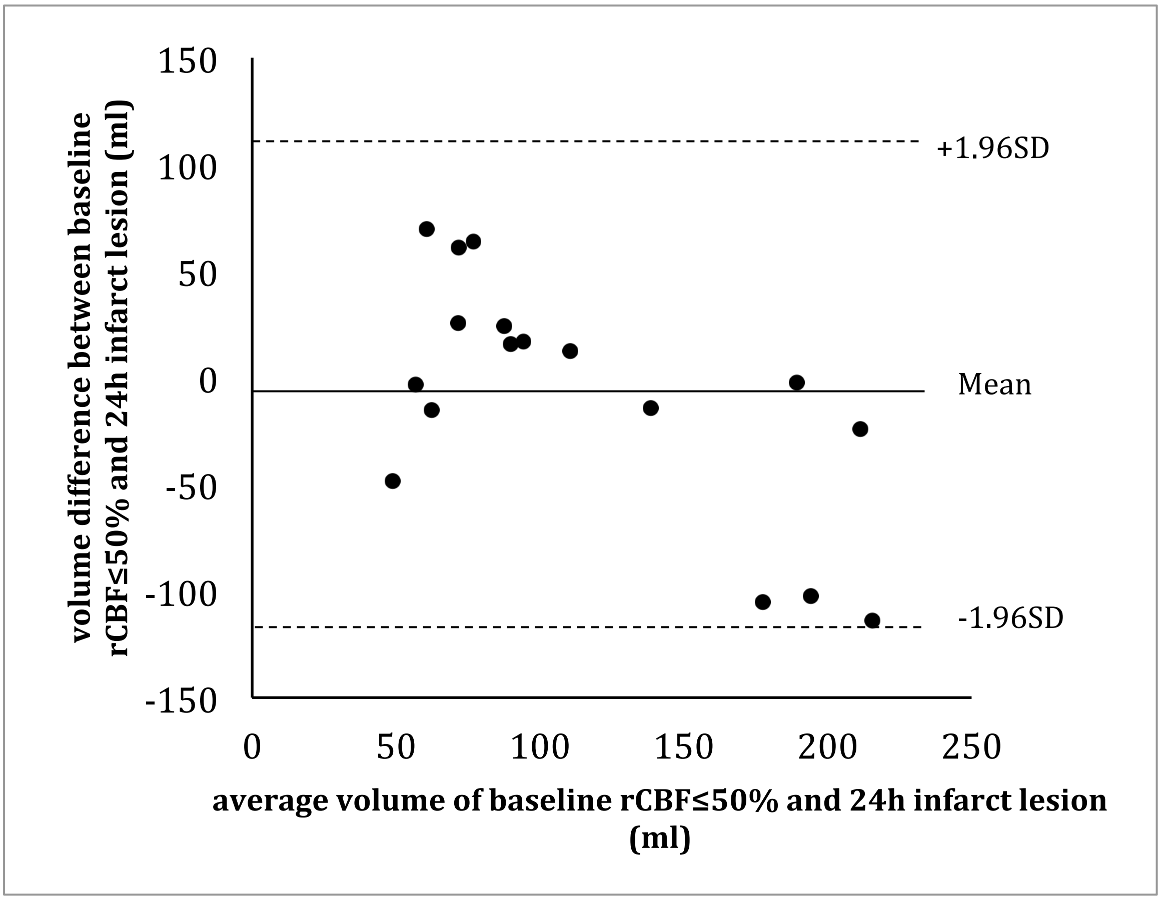

Figure II. Volumetric agreement of baseline rCBF≤50% (minimal-reperfusion group).

Figure III

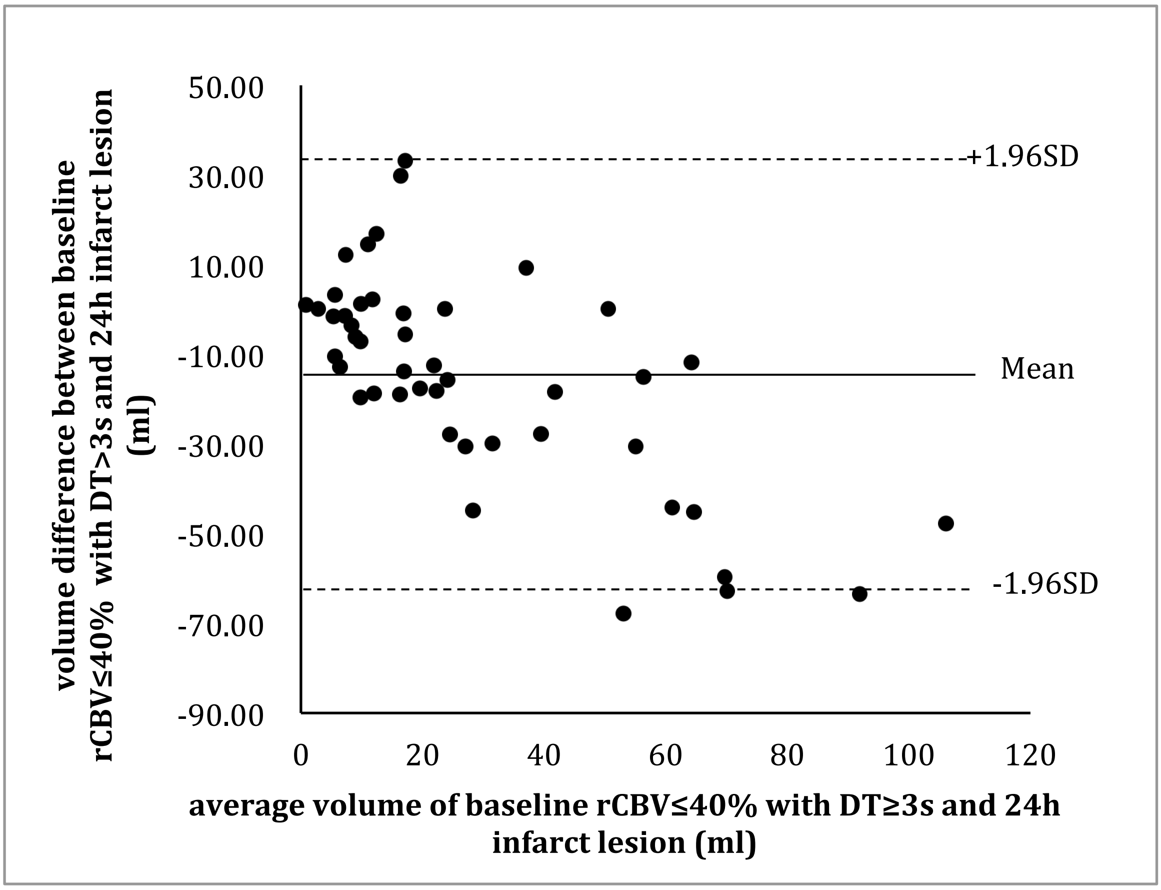

Figure III. Volumetric agreement of baseline rCBV≤40% within DT≥3s and 24h infarct lesion (major reperfusion group). The plot indicates the correlation between volume difference and average volume (r^2=0.38).

Figure IV

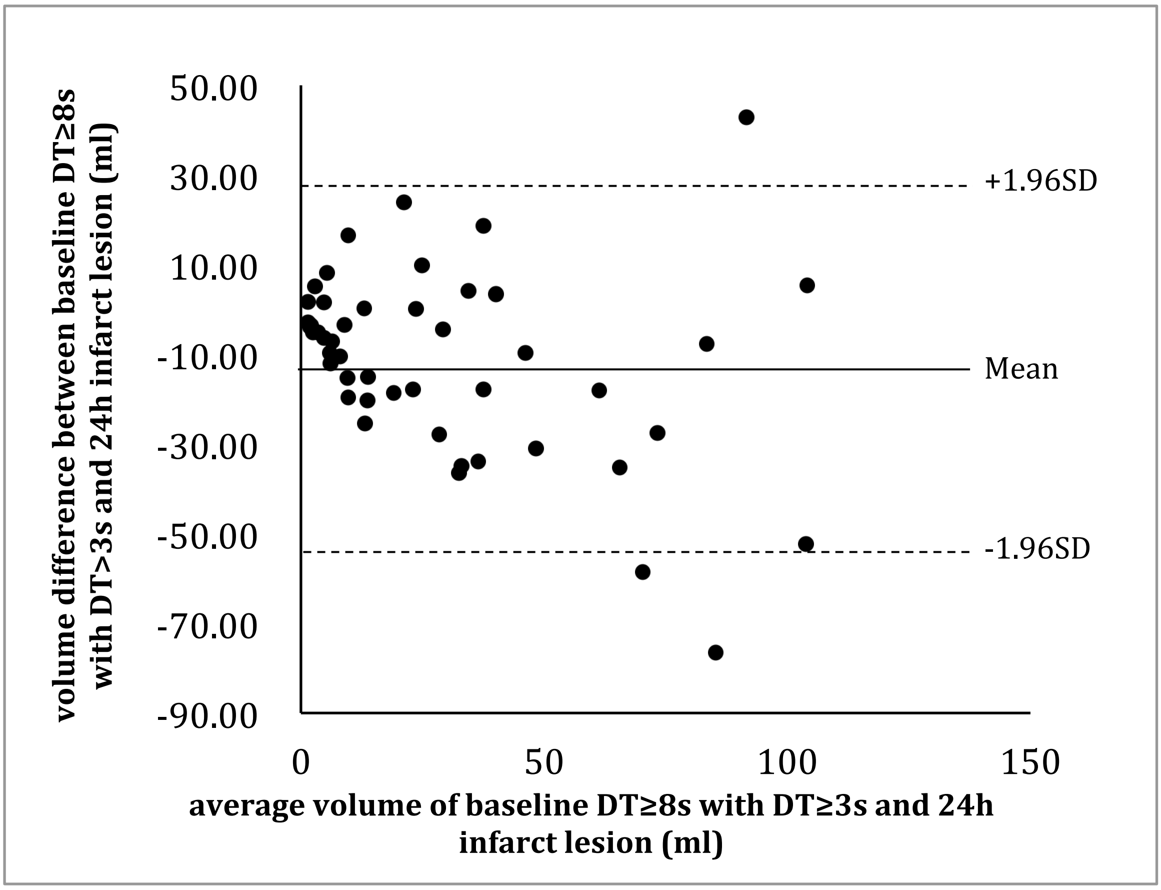

Figure IV. Volumetric agreement of baseline DT≥8s within DT≥3s and 24h infarct lesion (major reperfusion group).
